# Supplementary material for: How common is patient and public involvement (PPI)? Cross-sectional analysis of frequency of PPI reporting in health research papers and associations with methods, funding sources and other factors
Source: BMJ Open. 2022 May 24;12(5):e063356. doi: 10.1136/bmjopen-2022-063356 (PMC9131100; doi:10.1136/bmjopen-2022-063356)
Supplement: Author's manuscript [file bmjopen-2022-063356.draft_revisions.pdf]

BMJ Open is committed to open peer review. As part of this commitment we make the peer review history of every article we publish publicly available.

When an article is published we post the peer reviewers' comments and the authors' responses online. We also post the versions of the paper that were used during peer review. These are the versions that the peer review comments apply to.

The versions of the paper that follow are the versions that were submitted during the peer review process. They are not the versions of record or the final published versions. They should not be cited or distributed as the published version of this manuscript.

BMJ Open is an open access journal and the full, final, typeset and author-corrected version of record of the manuscript is available on our site with no access controls, subscription charges or pay-per-view fees (http://bmjopen.bmj.com).

If you have any questions on BMJ Open's open peer review process please email info.bmjopen@bmj.com

BMJ Open

How common is Patient and Public Involvement (PPI)? Cross-sectional analysis of frequency of PPI reporting in health research papers and associations with methods, funding sources, and other factors

Journal:	*BMJ Open*
Manuscript ID	bmjopen-2022-063356
Article Type:	Original research
Date Submitted by the Author:	29-Mar-2022
Complete List of Authors:	Lang, Iain; University of Exeter, PenARC<br>King, Angela; University of Exeter<br>Jenkins, Georgia; University of Exeter<br>Boddy, Kate; University of Exeter<br>Khan, Zohrah; University of Exeter<br>Liabo, Kristin ; University of Exeter Medical School, Patient and Public Involvement
Keywords:	STATISTICS & RESEARCH METHODS, MEDICAL ETHICS, EPIDEMIOLOGY

SCHOLARONE™
Manuscripts

I, the Submitting Author has the right to grant and does grant on behalf of all authors of the Work (as defined in the below author licence), an exclusive licence and/or a non-exclusive licence for contributions from authors who are: i) UK Crown employees; ii) where BMJ has agreed a CC-BY licence shall apply, and/or iii) in accordance with the terms applicable for US Federal Government officers or employees acting as part of their official duties; on a worldwide, perpetual, irrevocable, royalty-free basis to BMJ Publishing Group Ltd ("BMJ") its licensees and where the relevant Journal is co-owned by BMJ to the co-owners of the Journal, to publish the Work in this journal and any other BMJ products and to exploit all rights, as set out in our licence.

The Submitting Author accepts and understands that any supply made under these terms is made by BMJ to the Submitting Author unless you are acting as an employee on behalf of your employer or a postgraduate student of an affiliated institution which is paying any applicable article publishing charge ("APC") for Open Access articles. Where the Submitting Author wishes to make the Work available on an Open Access basis (and intends to pay the relevant APC), the terms of reuse of such Open Access shall be governed by a Creative Commons licence – details of these licences and which Creative Commons licence will apply to this Work are set out in our licence referred to above.

Other than as permitted in any relevant BMJ Author's Self Archiving Policies, I confirm this Work has not been accepted for publication elsewhere, is not being considered for publication elsewhere and does not duplicate material already published. I confirm all authors consent to publication of this Work and authorise the granting of this licence.

For peer review only

How common is Patient and Public Involvement (PPI)? Cross-sectional analysis of frequency of PPI reporting in health research papers and associations with methods, funding sources, and other factors

Authors: Iain Lang[1], Angela King (A.King6@exeter.ac.uk)[1], Georgia Jenkins (G.Jenkins2@exeter.ac.uk)[1], Kate Boddy (K.Boddy@exeter.ac.uk)[1], Zohrah Khan (zohrah.khan@nhs.net)[1], Kristin Liabo (K.Liabo@exeter.ac.uk)[1]

Corresponding author:

a. Iain Lang
b. College of Medicine and Health, University of Exeter, UK
c. i.lang@exeter.ac.uk

Affiliations:

1 College of Medicine and Health, University of Exeter.

For peer review only

Abstract

Objectives. Patient and public involvement (PPI) in research has become more widespread is required by some funders and publications but we know little about how common PPI. In this study we estimated the frequency of PPI inclusion in health research papers and analysed its relationship with research topics, methods, funding sources, and geographical regions.

Design. Cross-sectional

Methods. Our sample consisted of 3000 research papers published in 2020 in a general health-research journal (*BMJ Open*) that requires a statement on whether studies included PPI. We classified each paper as “included PPI” or “did not include PPI” and analysed the association of this classification with location (country or region of the world), methods used, research topic (journal section), and funding source. We used adjusted regression models to estimate incident rate ratios (IRRs) of PPI inclusion in relation to these differences.

Results. 618 (20.6%) of the papers in our sample included PPI. The proportion of papers including PPI varied in relation to location (from 44.5% (95% confidence interval (CI) 40.8 to 48.5) in papers from the UK to 3.4% (95% CI 1.5 to 5.3) in papers from China), method (from 38.6% (95% CI 27.1 to 50.1) of mixed-methods papers to 5.3% (95% CI -1.9 to 12.5) of simulation papers), topic (from 36.9% (95% CI 29.1 to 44.7) of papers on mental health to 3.4% (95% CI -1.3 to 8.2) of papers on medical education and training, and funding source (from 57.2% (95% CI 51.8% to 62.6%) in papers that received funding from the UK's National Institute for Health Research (NIHR) to 3.4% (95% CI 0.7% to 6.0%) in papers that received funding from a Chinese state funder).

Conclusions. Most research papers in our sample did not include PPI and PPI inclusion varied widely in relation to location, methods, topic, and funding source.

Word count (main text) = 3252

Keywords: patient and public involvement, PPI, patient engagement, stakeholder participation

For peer review only

Article Summary

Strengths and limitations of this study

- There has been little previous research on how often patient and public involvement (PPI) is included in health research papers and our sample (of 3000 papers) is much larger than that of previous studies
- As far as we are aware, this is the first study to analyse associations between PPI inclusion and other aspects of research papers such as topic and methods
- Our sample came from a single journal, but it is a general health- and medical-research journal that includes a broad range of topics and methods and requires authors to provide a statement on PPI inclusion
- We relied on published statements provided in research papers and cannot assess their accuracy

For peer review only

Background

Over the last two decades, the idea that patients and members of the public should be involved in health research has spread (1). The roots of this lie in the World Health Organization's Alma Alta Declaration (2), which stated that people have a "right and duty to participate individually and collectively in the planning and implementation of their healthcare." Those who promote patient and public involvement (PPI) in health research tend to do so for one or both of two reasons, one normative and one practical(3). The normative reason is that PPI is seen as making research more democratic and egalitarian and to move activity towards research done *with* or *by* people rather than *on* or *about* them (4). The practical reason is that PPI is seen as benefiting not only the process of research, for example by improving recruitment and retention of participants in clinical trials (5), but also the quality of research and the impact of research findings(6,7). As a means of implementing PPI in health research, some research funders now recommend or stipulate patient and public involvement (PPI) in the work they fund. This is done by some state funders, like the UK's National Institute for Health Research (8) and the Australian National Health and Medical Research Council (9), as well as by charitable funders such as the UK's Alzheimer's Society (10) and McPin Foundation (11).

However, reviews of PPI have found low levels of involvement and poor-quality reporting (12–16). Even in areas where we might expect high levels of patient involvement, such as trials of patient-oriented research or the development of patient-reported outcome measures, PPI is often poorly reported or absent (17,18). Research on attitudes towards PPI have identified reluctance and resistance from health researchers grounded in perceptions of the costs involved (e.g., in terms of researcher workload,(19), uncertainties about how to do it (20) or report it (21), and difficulties related to issues such as tokenism, box-ticking, and the role of PPI as a form of governance (22–26) .

To date, research on PPI has been almost entirely qualitative and we lack important information on how widespread PPI is within health research and in what circumstances researchers are more or less likely to include it. Our aim in this study was to provide some of this information. We used data on PPI in papers published in BMJ Open, an open-access journal in which PPI reporting is obligatory and authors are required to include a statement about PPI even if they did not include PPI in their study. We looked at all the PPI statements in all research papers published in BMJ Open in a twelve-month period. We analysed, first, how common PPI inclusion was and, second, whether and to what extent PPI varied in relation to location, method, topic, and funding source.

Methods

We used ProQuest, an online bibliographic database (www.proquest.com), to identify all original research papers published in BMJ Open during the twelve months from 1st January 2020 to 31st December 2020 (n=3000). The data for our study were the full texts of papers plus accompanying metadata (information on each paper detailing date of publication, document IDs, journal section, and so on). We used a custom-written Perl (27) script to read the data file, extract relevant information, and assemble our analysis dataset.

For each paper, we extracted:

- *First author name*, for ease of reference and reporting

For peer review only

- the *URL* that linked directly to the online version of the paper using its digital object identifier (DOI) and which also constituted a unique identifier for each paper
- *Patient and public involvement statement*: full text of the statement made in the paper (as part of the main text) or following the paper (alongside the acknowledgments)
- *Location* (country or region) from which the paper came, based on the country of the primary affiliation of the first author. Many countries were associated with only a small number of papers and for this reason, as well as for ease and clarity of reporting, we bundled all countries with fewer than 100 associated papers into regions. This left us with seven individual countries and six regions
- *Method,* based on our categorization of words in the title, from which we identified 18 terms that allowed us to assign each paper to at least one category (systematic review, protocol, trial, etc.) in relation to methods. Some papers had more than one method associated with them because they contained multiple terms, e.g., both protocol and trial.
- *Research Topic,* based on the section of the journal to which the paper was assigned. Most of these related to substantive topics (Public Health, Cardiovascular Medicine, Nursing, etc.) and some relate to methods (Epidemiology, Qualitative research, Health Economics, etc.) but each paper is assigned to only one section. The papers in our sample fell into 61 sections but many of these had only small numbers of papers in them. For reporting purposes, below, we provide details of research topics from the 19 sections containing at least 50 papers.
- *Funding source*, extracted from the Funding section of each paper. We identified all funders associated with each paper but for reporting purposes we only provide details of those with at least 25 papers related to them: there were twelve of these. These more common funders were associated with approximately one-third of the papers in our sample.

We looked at four *exposure variables* based on this extracted information: location, method, research topic, and funding source. Our aim was to understand whether papers that differed in relation to these categories (differences in location, differences in methods, and so on) were also likely to differ in terms of including PPI.

We based our *outcome variable* on our interpretation of the PPI statement and categorized each paper as either "included PPI" or "did not include PPI". We used the Perl script to find phrases commonly used in PPI statements, such as "no patients involved", "patients and public were not involved", and "patients and/or the public were not invited". Based on this, the script assigned 1797 of the 3000 papers to the "did not include PPI" group. This assignation helped us reach our final decision on the group in which we should put each paper but did not constitute our decision: we read through each statement to check that we agreed with the categorization.

Deciding what does and does not constitute PPI is conceptually difficult, especially when only limited information – short statements provided by authors – is available. We classed papers as "included PPI" if any form of involvement by one or more non-professional researchers, whether as individuals or in the form of a group, was claimed at any stage of the study. We classed as "did not include PPI" papers in which:

- PPI was explicitly omitted (e.g., "Patients and the public were not involved in this research study" (28)

For peer review only

- Patient input was taken from previous literature or previous studies (e.g., “Previous studies about patient experiences in OLP and placebo trials of irritable bowel syndrome were consulted for this study to include patient experiences” (29))
- PPI-type input was provided by experts whose primary role was not to provide knowledge from lived experience or public oversight of the study (e.g., “The indication, research questions and study endpoint outcome measures were selected based on the authors’ expert understanding in the care of affected patients, their needs and therapy preferences, without direct communication of the study design to patients”(30).

One of the authors (IL) took responsibility for reading all the PPI statements, correcting the script-based assignments where they were wrong, and making a final decision on the categorization of each paper. IL and two other authors (KB and KL) did initial calibration for this decision-making in concert: all three of us discussed several borderline cases and arrived at a consensus on how to classify these. In 130 of the papers in our dataset we could not find a statement or any other information about PPI and we coded these as not including PPI.

Analysis

We analysed our data using Stata/SE 17.0 and Microsoft Excel. For each exposure category (location, method, research topic, funder) we calculated descriptive statistics relating to the number of paper assigned to each category and the mean level of PPI in each. For example, in relation to methods, did papers that reported trials, evaluations, or systematic reviews differ in terms of how likely they were to include PPI? What about papers that concerned surgery compared to those about anaesthetics or general practice? For the groupings within each category we estimated, with 95% confidence intervals, the proportion of papers in which the authors reported the inclusion of PPI.

In the regression models of which we report the results below, we used Stata to estimate the relative risk and confidence intervals of having PPI by means of generalized linear models with a modified Poisson approach and robust error variances (31). To account for potential confounding, we adjusted our models for location, method, and topic for each of the other exposure variables (location/ method/ topic/ funding source). When the outcome was funding source we adjusted for method and topic but not for location, because location and funding source were often overlapping. For example, of the 178 papers receiving funding from Chinese state funders, 174 (97.8%) came from China – see additional analyses below.

The highest and lowest levels of PPI inclusion were associated, respectively, with papers receiving funding from two national government funders, the UK’s NIHR and Chinese state funders. To provide insight into the relationships with PPI inclusion of being based in a specific country and of being supported by the state funder in that country, we conducted additional analyses to look at the differences in PPI inclusion levels in UK papers that were and that were not funded by NIHR, and in Chinese papers that were and were not funded by Chinese state funders.

Ethics approval

Since this study used publicly available data and involved neither human nor animal subjects, we did not submit it for, and it has not received, formal ethics approval.

For peer review only

Patient and Public Involvement

One of our co-authors, AK, is not a professional researcher but has experience of being involved in research as a patient and carer. She joined the study after inception and was involved in data analysis and interpretation and in writing the manuscript. From the perspective of the professional co-authors, AK's most important contribution has been to challenge us on how we have described PPI throughout the paper. She drew our attention to issues around language and power in an early draft and because of this we changed the way in which we described PPI throughout the paper.

Results

Of the 3000 papers in our sample, we identified 618 (20.6%, 95% CI 19.1% to 22.0%) as having included PPI. The tables and figures below show the distribution of these papers in relation to the four types of difference we examined: location (Table 1), method (Table 2), research topic (Table 3), and funder (Table 4). Each table shows the unadjusted estimates of mean PPI in each category, ordered from greatest to lowest:

- in relation to *location* (Table 1), the likelihood of PPI inclusion ranged from 44.5% (95% CI 40.5% to 48.5%) in the UK to 3.4% (95% CI 1.5% to 5.3%) in China.
- in relation to *method* (Table 2), the likelihood of PPI inclusion ranged from 38.6% (95% CI 27.1% to 50.1%) in mixed-methods papers to 5.3% (95% CI -1.9% to 12.5%) in simulation papers.
- in relation to *research topic* (Table 3), the likelihood of PPI inclusion ranged from 36.9% (95% CI 29.1% to 44.7%) in papers on mental health to 3.4% (95% CI -1.3% to 8.2%) of papers on medical education and training.
- in relation to *funding source* (Table 4), the likelihood of PPI inclusion ranged from 57.2% (95% CI 51.8% to 62.6%) in papers that received funding from NIHR to 3.4% (95% CI 0.7% to 6.0%) in papers that received funding from a Chinese state funder.

Papers that did not receive funding from any of the major funders (that is, those included in Table 4) (n=2028) had a mean level of PPI inclusion of 14.9% (95% confidence interval 13.4% to 16.5%), which is statistically significantly lower than the mean level of PPI inclusion of the whole dataset.

Figures 1, 2, 3, and 4 show the relative risks and confidence intervals of each category estimated using an adjusted generalized linear model as described above for location, method, research topic, and funding source, respectively.

For peer review only

Table 1. Number of papers published in BMJ Open in 2020 by location, and proportion of these with PPI

Location	n	% of total	Mean %PPI (95% CI)
UK	591	19.7	44.5 (40.5 to 48.5)
Canada	195	6.5	30.8 (24.3 to 37.3)
Australia/NZ	269	9.0	21.9 (17.0 to 26.9)
France	118	3.9	21.2 (13.8 to 28.6)
Netherlands	120	4.0	20.8 (13.5 to 28.1)
Germany	101	3.4	16.8 (9.5 to 24.2)
USA	227	7.6	16.3 (11.5 to 21.1)
Europe (other)	501	16.7	15.5 (12.4 to 18.7)
Central and South America	59	2.0	11.3 (3.9 to 18.7)
Central and South Asia	72	2.4	10.2 (2.4 to 18.0)
Africa and West Asia	163	5.4	8.0 (3.8 to 12.1)
East and South-east Asia	232	7.7	6.4 (3.3 to 9.6)
China	352	11.7	3.4 (1.5 to 5.3)
	3000	100.0	20.6 (19.1 to 22.0)

Note that location here means each country with ≥100 papers and otherwise means region

Table 2. Number of papers published in BMJ Open in 2020 by method used, and proportion of these with PPI

Method	n	% of total	Mean %PPI (95% CI)
Mixed methods	70	2.3	38.6 (27.1 to 50.1)
Implementation	65	2.2	35.4 (23.7 to 47.1)
Trial	551	18.4	34.8 (30.9 to 38.8)
Qualitative	260	8.7	34.2 (28.4 to 40.0)
Evaluation	115	3.8	33.0 (24.4 to 41.7)
Pilot study	42	1.4	31.0 (16.8 to 45.1)
Development	118	3.9	29.7 (21.3 to 37.9)
Protocol	1181	39.3	28.9 (26.3 to 31.5)
Health economics	91	3.0	25.3 (16.3 to 34.3)
Survey	158	5.3	17.7 (11.7 to 23.7)
Observational	220	7.3	15.9 (11.1 to 20.8)
Cohort	637	21.2	14.6 (11.9 to 17.3)
Scoping	125	4.2	13.6 (7.6 to 19.6)
Time series	25	0.8	12.0 (-1.0 to 25.0)
Systematic review	376	12.5	9.6 (6.6 to 12.6)
Cross-sectional	464	15.5	9.3 (6.6 to 11.9)
Meta-analysis	265	8.9	8.3 (5.0 to 11.6)
Simulation	38	1.3	5.3 (-1.9 to 12.5)

Note that total n and % of papers here each sum to more than the total number of papers or to >100% because some have more than one designation – e.g., both protocol and trial

For peer review only

Table 3. Number of papers published in BMJ Open in 2020 in each research topic with ≥50 papers, and proportion of these with PPI

Research topic	n	% of total	Mean %PPI (95% CI)
Mental health	149	5.0	36.9 (29.1 to 44.7)
Qualitative research	51	1.7	35.3 (22.0 to 48.5)
Health services research	168	5.6	31.0 (23.9 to 38.0)
Neurology	82	2.7	29.3 (19.4 to 39.2)
Surgery	102	3.4	28.4 (19.6 to 37.2)
Rehabilitation medicine	65	2.2	26.2 (15.4 to 36.9)
Oncology	88	2.9	25.0 (15.9 to 34.1)
Cardiovascular medicine	120	4.0	24.2 (16.5 to 31.9)
Paediatrics	91	3.0	23.1 (14.4 to 31.8)
Diabetes and endocrinology	85	2.8	22.4 (13.4 to 31.3)
Obstetrics and gynaecology	98	3.3	21.4 (13.3 to 29.6)
Emergency medicine	66	2.2	21.2 (11.3 to 31.2)
General practice / Family practice	94	3.1	20.2 (12.0 to 28.4)
Public health	366	12.2	16.1 (12.3 to 19.9)
Infectious diseases	79	2.6	15.2 (7.2 to 23.2)
Global health	97	3.2	13.4 (6.6 to 20.2)
Health economics	61	2.0	6.6 (0.3 to 12.8)
Epidemiology	241	8.0	6.2 (3.2 to 9.3)
Medical education and training	58	1.9	3.4 (-1.3 to 8.2)
	1261	71.8	

Table 4. Number of papers published in BMJ Open in 2020 by funding source for funders named in ≥25 papers, and proportion of these with PPI

Funder	n	% of total	Mean %PPI (95% CI)
NIHR	320	10.7	57.2 (51.8 to 62.6)
CIHR	90	3.0	41.1 (30.9 to 51.3)
ZonMw	25	0.8	40.0 (20.4 to 59.6)
MRC	58	1.9	39.7 (27.0 to 52.4)
ESRC	41	1.4	29.3 (15.2 to 43.4)
Wellcome Trust	103	3.4	28.2 (19.4 to 36.9)
NHMRC	111	3.7	27.9 (19.5 to 36.3)
NIH	81	2.7	27.2 (17.4 to 36.9)
EU	69	2.3	23.2 (13.2 to 33.2)
Carlos III	30	1.0	13.3 (1.0 to 25.7)
Gates Foundation	33	1.1	12.1 (0.8 to 23.4)
Chinese	178	5.9	3.4 (0.7 to 6.0)

Note: NIHR = National Institute of Health Research (UK); ZonMw = The Netherlands Organisation for Health Research and Development; CIHR = Canadian Institutes of Health Research; MRC = Medical Research Council (UK); ESRC = Economic and Social Research Council (UK); NHMRC = National Health and Medical Research Council (Australia); Wellcome = The Wellcome Trust (UK); EU = European Union funding; NIH = US National Institutes of Health; Carlos III = Instituto de Salud Carlos III (Spain); Gates = The Bill and Melinda Gates Foundation (USA); Chinese = Key Research or other Chinese state funder, e.g. National Science Foundation of China)

For peer review only

Additional analyses

Of the 320 papers whose authors reported receiving funding from the UK's NIHR, 272 (85.0%) came from the UK. Those 272 papers represent 46.0% of the 591 UK papers in our sample, and 59.9% (95% CI 54.1% to 65.7%) of them included PPI. Of the 319 (54.0%) of UK papers whose authors did not report receiving NIHR funding, 31.3% (95% CI 26.2% to 36.5%) included PPI. For UK papers, receipt of NIHR funding was associated with increased PPI inclusion.

Of the 178 papers whose authors reported receiving funding from Chinese state funders, 174 (97.8%) came from China. Those 174 papers represent 49.3% of the 352 Chinese papers in our sample, and 3.4% (95% CI 0.7% to 6.2%) of them included PPI. Of the 178 (50.6%) of Chinese papers whose authors did not report receiving Chinese state funding, 3.4% (95% CI 0.7% to 6.0%) included PPI. For Chinese papers, receipt of Chinese state funding was not associated with any difference in PPU inclusion.

Discussion

We had two aims in this study: to assess how common PPI is in a sample of published health research papers, and to measure how much variation there is in PPI inclusion according to known differences between the papers. We addressed these aims by looking the PPI statements included in 3000 papers published in general health and medical research journal where authors are required to include a statement about PPI, BMJ Open. In relation to our first aim, we found that approximately one in five (20.6%) of the papers in our sample reported including PPI. In relation to the second, we found marked variations in PPI inclusion across the categories we looked at. For example, papers from the UK were more than ten times likelier to include PPI than those from China, and there were also marked differences in relation to each of method, topic, and funding source.

These large differences in PPI inclusion may indicate not only what influences PPI but of what might be done to increase it. It seems clear that authors in some research fields, for example, are less persuaded of the importance of PPI and targeting of journals and researchers in those fields might increase involvement. Our findings also suggest that funders can make a difference in PPI. The funding source we found to be associated with the highest level of PPI inclusion was the UK's National Institute for Health Research (NIHR), which has for several years included in its application process a section in which applicants may either apply for funding to include PPI or justify why they are not including it. We note, at the same time, that NIHR lacks a single, unequivocal policy statement about PPI (32).

Previous work has reviewed PPI reporting in specific topics such as dementia (13,33), patient-oriented-research trials (17), and surgical trials (14). Although the authors of these studies did not attempt to measure the proportion of published papers involving PPI, as we have done here, they uniformly identify few studies reporting PPI within the large literatures in which they were interested; a study of clinical trials published in nursing journals failed to find any evidence of PPI (34). We know of only one recent study on the prevalence of PPI in general medical research, which, in a smaller sample, found that 11% of papers reported PPI inclusion (35). We are unaware of any other studies on the prevalence of PPI inclusion or of factors associated with it.

Our study has a couple of noteworthy shortcomings. The first of these relates to PPI statements. We took authors' PPI statements at face value: if the statement said that PPI took place then we took this to be the case, even though there is evidence that what researchers say they will do and what they actually do, in relation to PPI, varies (36). Authors

For peer review only

may have exaggerated, underplayed, or otherwise misreported the extent of PPI in their studies but we note the conclusion of a previous study: that apparently low levels of PPI inclusion are not simply due to under-reporting (35). We also had no way of telling whether PPI statements reflect the experience of involvement of the individuals or groups providing the PPI.

Another aspect of this is that PPI may be understood differently in different places and, as a result, reported differently. We found PPI is more common in health research from the UK than elsewhere. The development and institutionalization of PPI in the UK can be seen as the outcome of specific national socio-political events and trends. As a result, the term "patient and public involvement" has resonances in UK health research that may be different or absent elsewhere. In other settings there may be other ways of achieving or addressing the concerns that PPI is seen as addressing (for example, through working with patient-interest organizations rather than with individual patient representatives) and a focus on PPI may overlook these. Although not referring to health research, Zhu (37) describes how patient involvement and participation in Chinese hospitals is influenced by factors including level of civil society engagement, moves towards marketisation, and individuals' cultural capital. Possibilities for and meanings of PPI may vary widely in relation to both national and sub-national differences.

The second shortcoming relates to our sample. We used a purposeful sample of papers from a single journal, BMJ Open – if this was a clinical study we might call it a case series. We used BMJ Open because, as far as we know, it is the only journal that (a) has a written policy requiring papers to include a PPI statement *and* (b) is open access *and* (c) is not discipline- or method-specific and publishes papers across a range of health-related topics and using a range of methods. However, peer-reviewed journals are, by definition, selective in the papers in they publish. BMJ Open publishes many study protocols and many papers by authors based in the UK. The journal's policy regarding PPI may encourage authors to submit papers that include PPI and discourage them from submitting those that lack it. These characteristics may influence the topics and methods submitted to the journal as well as actions concerning PPI.

We believe there is scope for further research on the issues we have begun to explore here. This might include work on the quality of PPI reporting, perhaps based on established criteria (of note, the GRIPP2 reporting checklists (38) – probably the best-known guidelines for reporting PPI – were cited only 12 times in our sample); replication of our analyses using a sample of papers taken from elsewhere; and qualitative analysis of the statements made both by authors who report PPI and those who report none. Our dichotomisation of statements into "included PPI" or "did not include PPI" ignores any issues of quality or extent of involvement and this topic merits investigation. Our analysis has looked only at associations between PPI involvement and broad categories such as country of origin or funding body and not micro-level differences such as attitudes, policies, or beliefs at the level of research organisations, research groups, or individual researchers. We will need different methods to study such influences on how and why PPI in health research occurs – and why it does not.

Contributorship Statement: Concept and design, statistical analysis, and drafting of the manuscript: IL. Acquisition, analysis, or interpretation of the data, critical revision of the manuscript for important intellectual content, approval of final version, and agreement to be accountable for all aspects of the work: IL, AK, GJ, KB, ZK, KL.

Competing interests: None declared

For peer review only - http://bmjopen.bmj.com/site/about/guidelines.xhtml

Funding: This report is independent research supported by the UK National Institute for Health Research Applied Research Collaboration South West Peninsula (Grant Reference Number: NIHR200167). The views expressed in this publication are those of the author(s) and not necessarily those of the National Institute for Health Research or the UK Department of Health and Social Care.

Data sharing statement: Data for this study came from an open-source journal and are freely available online

References

1. Hoddinott P, Pollock A, O'Cathain A, Boyer I, Taylor J, MacDonald C, et al. How to incorporate patient and public perspectives into the design and conduct of research. F1000Research. 2018;7:752.

2. World Health Organisation. Declaration of Alma Ata: report of the International Conference on Primary Health Care. In Geneva; 1978.

3. Ives J, Damery S, Redwod S. PPI, paradoxes and Plato: who's sailing the ship? J Med Ethics. 2013 Mar;39(3):181–5.

4. Heron J, Reason P. Extending Epistemology within a Co-operative Inquiry. In: The SAGE Handbook of Action Research [Internet]. 1 Oliver's Yard, 55 City Road, London England EC1Y 1SP United Kingdom: SAGE Publications Ltd; 2008 [cited 2022 Mar 11]. p. 366–80. Available from: http://methods.sagepub.com/book/the-sage-handbook-of-action-research/d32.xml

5. Crocker JC, Ricci-Cabello I, Parker A, Hirst JA, Chant A, Petit-Zeman S, et al. Impact of patient and public involvement on enrolment and retention in clinical trials: systematic review and meta-analysis. BMJ. 2018 Nov 28;363:k4738.

6. Staley K. Exploring impact: public involvement in NHS, public health and social care research. Eastleigh, England: INVOLVE; 2009.

7. Evans D, Coad J, Cottrell K, Dalrymple J, Davies R, Donald C, et al. Public involvement in research: assessing impact through a realist evaluation [Internet]. Southampton (UK): NIHR Journals Library; 2014 [cited 2022 Mar 11]. (Health Services and Delivery Research). Available from: http://www.ncbi.nlm.nih.gov/books/NBK260168/

8. UK Public Involvement Standards Development Partnership. UK Standards for Public Involvement: Better public involvement for better health and social care research [Internet]. [cited 2022 Mar 18]. Available from: https://drive.google.com/file/d/1U-IJNJCfFepaAOruEhzz1TdLvAcHTt2Q/view

9. Consumers Health Forum of Australia. Statement on consumer and community involvement in health and medical research. National Health and Medical Research Council; 2016.

10. Alzheimer's Society. Alzheimer's Society Research Delivery Plan 2018/2022 [Internet]. 2017 [cited 2022 Mar 14]. Available from: https://www.alzheimers.org.uk/sites/default/files/2019-05/AS_ResearchDeliveryPlan_2019_v6final.pdf

11. McPin Foundation. Patient & Public Involvement at the McPin Foundation: An overview. Version 1.1, July 2017 [Internet]. 2017 [cited 2022 Mar 14]. Available from: https://mcpin.org/wp-content/uploads/McPin-PPI-slides-v1.1-july-2017.pdf

12. Cook N, Siddiqi N, Twiddy M, Kenyon R. Patient and public involvement in health research in low and middle-income countries: a systematic review. BMJ Open. 2019 May 1;9(5):e026514.

13. Miah J, Dawes P, Edwards S, Leroi I, Starling B, Parsons S. Patient and public involvement in dementia research in the European Union: a scoping review. BMC Geriatr. 2019 Aug 14;19(1):220.

For peer review only

14. Jones EL, Williams-Yesson BA, Hackett RC, Staniszewska SH, Evans D, Francis NK. Quality of Reporting on Patient and Public Involvement Within Surgical Research: A Systematic Review. Ann Surg. 2015 Feb;261(2):243–50.

15. Muir R, Carlini JJ, Harbeck EL, Gillespie BM, Tuffaha HW, Walker RM, et al. Patient involvement in surgical wound care research: A scoping review. Int Wound J. 2020;17(5):1462–82.

16. Patel VA, Shelswell J, Hillyard N, Pavitt S, Barber SK. A study of the reporting of patient and public involvement and engagement (PPIE) in orthodontic research. J Orthod. 2021 Mar 1;48(1):42–51.

17. Fergusson D, Monfaredi Z, Pussegoda K, Garritty C, Lyddiatt A, Shea B, et al. The prevalence of patient engagement in published trials: a systematic review. Res Involv Engagem. 2018 May 22;4(1):17.

18. Wiering B, de Boer D, Delnoij D. Patient involvement in the development of patient-reported outcome measures: a scoping review. Health Expect. 2017;20(1):11–23.

19. Baldwin JN, Napier S, Neville S, Wright-St Clair VA. Impacts of older people's patient and public involvement in health and social care research: a systematic review. Age Ageing. 2018 Nov 1;47(6):801–9.

20. Domecq JP, Prutsky G, Elraiyah T, Wang Z, Nabhan M, Shippee N, et al. Patient engagement in research: a systematic review. BMC Health Serv Res. 2014 Feb 26;14(1):89.

21. Price A, Albarqouni L, Kirkpatrick J, Clarke M, Liew SM, Roberts N, et al. Patient and public involvement in the design of clinical trials: An overview of systematic reviews. J Eval Clin Pract. 2018;24(1):240–53.

22. Komporozos-Athanasiou A, Renedo A, McKevitt C. Citizen Participation in Neoliberal Times. Sociol Res Online. 2019 Sep 1;24(3):370–5.

23. Komporozos-Athanasiou A, Paylor J, Mckevitt C. Governing Researchers through Public Involvement. J Soc Policy. 2021 Apr 16;1–16.

24. Sangill C, Buus N, Hybholt L, Berring LL. Service user's actual involvement in mental health research practices: A scoping review. Int J Ment Health Nurs. 2019;28(4):798–815.

25. Boaz A, Biri D, McKevitt C. Rethinking the relationship between science and society: Has there been a shift in attitudes to Patient and Public Involvement and Public Engagement in Science in the United Kingdom? Health Expect. 2016;19(3):592–601.

26. Lemonsky F. Service user involvement in research. Lancet Psychiatry. 2015 Sep 1;2(9):780.

27. Wall L, Christiansen T, Schwartz R. Programming Perl. 3rd edition. USA: O'Reilly Media, Inc; 2000. 670 p.

28. Alahdab F, Halvorsen AJ, Mandrekar JN, Vaa BE, Montori VM, West CP, et al. How do we assess resilience and grit among internal medicine residents at the Mayo Clinic? A longitudinal validity study including correlations with medical knowledge, professionalism and clinical performance. BMJ Open. 2020 Dec 1;10(12):e040699.

29. Nascimento AF, Gaab J, Kirsch I, Kossowsky J, Meyer A, Locher C. Open-label placebo treatment of women with premenstrual syndrome: study protocol of a randomised controlled trial. BMJ Open. 2020 Feb 1;10(2):e032868.

30. Vincenzi B, Trower M, Duggal A, Guglielmini P, Harris P, Jackson D, et al. Neurokinin-1 antagonist orvepitant for EGFRI-induced pruritus in patients with cancer: a randomised, placebo-controlled phase II trial. BMJ Open. 2020 Feb 1;10(2):e030114.

31. Zou G. A Modified Poisson Regression Approach to Prospective Studies with Binary Data. Am J Epidemiol. 2004 Apr 1;159(7):702–6.

For peer review only

32. Russell J, Greenhalgh T, Taylor M. Patient and public involvement in NIHR research 2006-2019: policy intentions, progress and themes. :37.

33. Bethell J, Commisso E, Rostad HM, Puts M, Babineau J, Grinbergs-Saull A, et al. Patient engagement in research related to dementia: A scoping review. Dementia. 2018 Nov 1;17(8):944–75.

34. Gray R, Brasier C, Zirnsak T-M, Ng AH. Reporting of patient and public involvement and engagement (PPIE) in clinical trials published in nursing science journals: a descriptive study. Res Involv Engagem. 2021 Dec 14;7(1):88.

35. Price A, Schroter S, Snow R, Hicks M, Harmston R, Staniszewska S, et al. Frequency of reporting on patient and public involvement (PPI) in research studies published in a general medical journal: a descriptive study. BMJ Open. 2018 Mar 1;8(3):e020452.

36. Blackburn S, McLachlan S, Jowett S, Kinghorn P, Gill P, Higginbottom A, et al. The extent, quality and impact of patient and public involvement in primary care research: a mixed methods study. Res Involv Engagem. 2018 May 24;4(1):16.

37. Zhu J. The Meaning of Patient Involvement and Participation in Chinese Hospitals [Internet]. University of Edinburgh; 2021. Available from: https://era.ed.ac.uk/bitstream/handle/1842/37848/Zhu2021.pdf

38. Staniszewska S, Brett J, Simera I, Seers K, Mockford C, Goodlad S, et al. GRIPP2 reporting checklists: tools to improve reporting of patient and public involvement in research. BMJ. 2017 Aug 2;358:j3453.

Figure 1. Relative risk of PPI (incidence-rate-ratio) in studies by location compared to most common to UK, with 95% confidence intervals, adjusted by method, topic, and funder

Note: Incidence-rate ratios (y-axis) are on a logarithmic scale (base 10). Location here means each country with ≥100 papers and otherwise means region

Figure 2. Relative risk (incidence-rate ratio) of PPI in studies by method, with 95% confidence intervals, adjusted by location, topic, and funder

Note: Incidence-rate ratios (y-axis) are on a logarithmic scale (base 10)

Figure 3. Relative risk (incidence-rate ratio) of PPI in studies by topic compared to Health Service Research, with 95% confidence intervals, adjusted by location, method, and funder

Note: Incidence-rate ratios (y-axis) are on a logarithmic scale (base 10)

For peer review only

Figure 4. Relative risk (incidence-rate ratio) of PPI in studies by funding source compared to mean level, with 95% confidence intervals, adjusted by method and topic

Note: NIHR = National Institute of Health Research (UK); ZonMw = The Netherlands Organisation for Health Research and Development; CIHR = Canadian Institutes of Health Research; MRC = Medical Research Council (UK); ESRC = Economic and Social Research Council (UK); NHMRC = National Health and Medical Research Council (Australia); Wellcome = The Wellcome Trust (UK); EU = European Union funding; NIH = US National Institutes of Health; Carlos III = Instituto de Salud Carlos III (Spain); Gates = The Bill and Melinda Gates Foundation (USA); Chinese = Key Research or other Chinese state funder, e.g. National Science Foundation of China). Incidence-rate ratios (y-axis) are on a logarithmic scale (base 10)

For peer review only

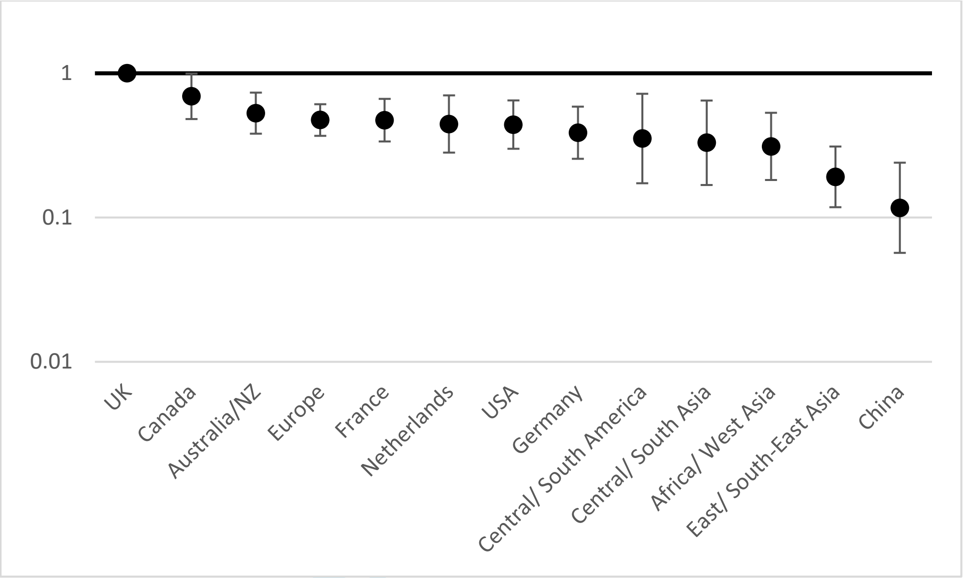

For peer review only

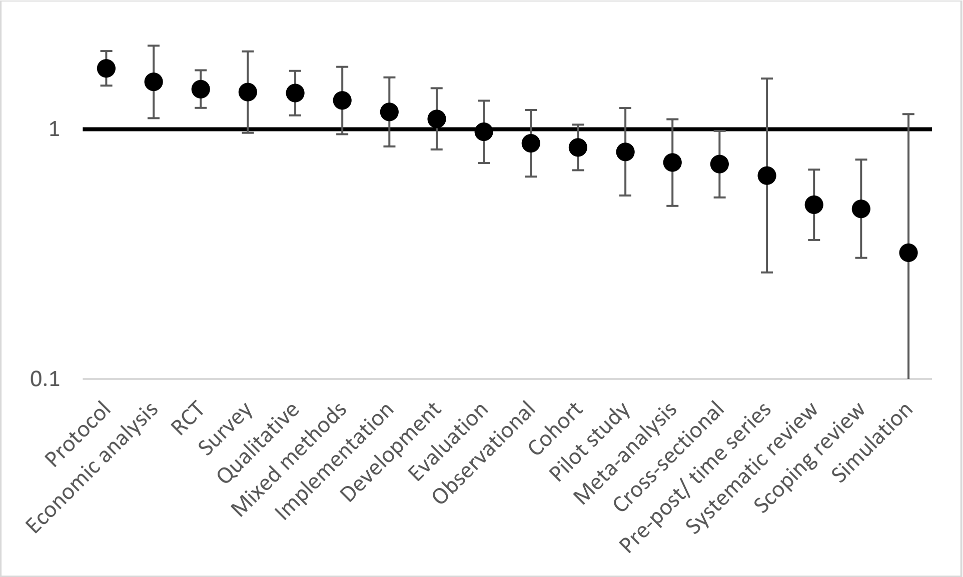

For peer review only

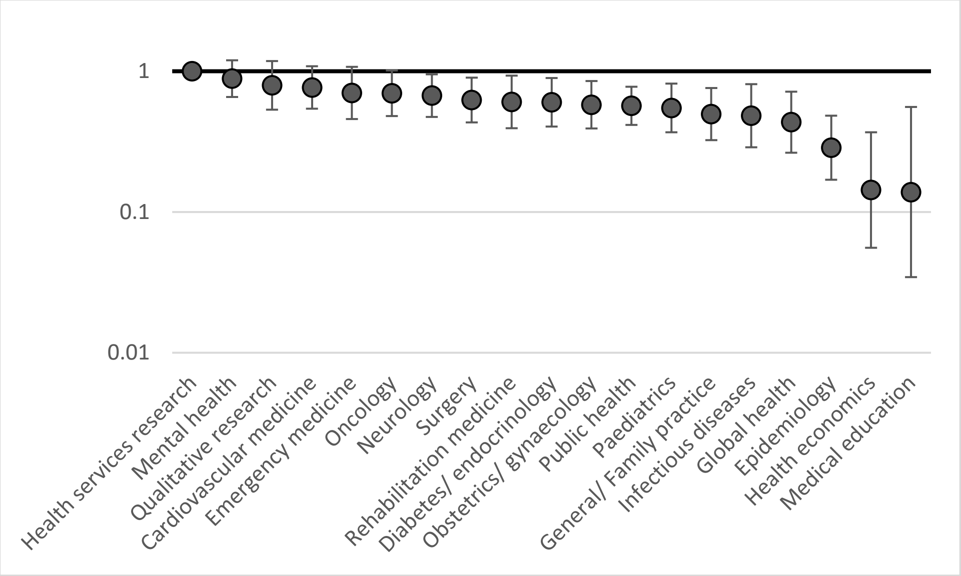
1
0.1
0.01
Health services research
Mental health
Qualitative research
Cardiovascular medicine
Emergency medicine
Oncology
Neurology
Surgery
Rehabilitation medicine
Diabetes/ endocrinology
Obstetrics/ gynaecology
Public health
Paediatrics
General/ Family practice
Infectious diseases
Global health
Epidemiology
Health economics
Medical education

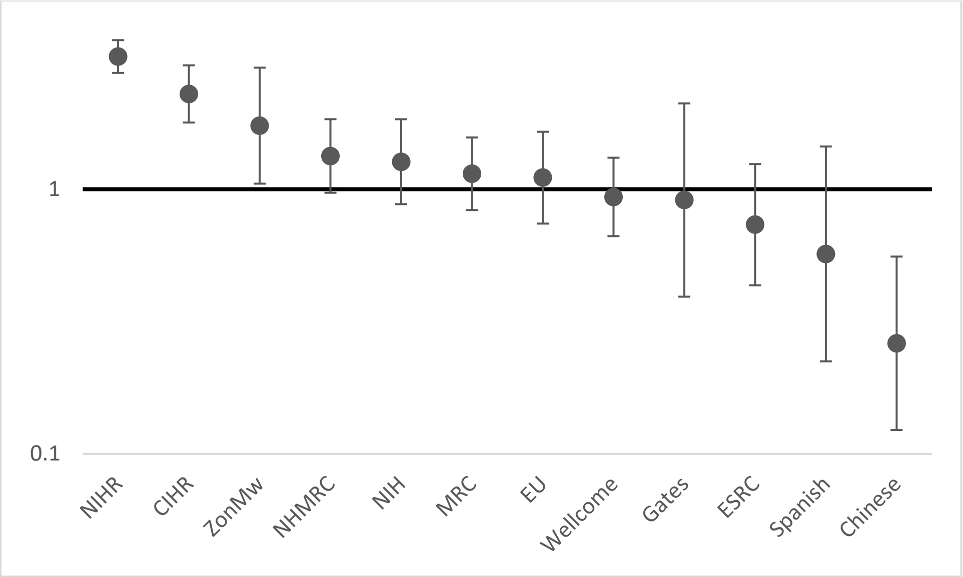
1
0.1
NIHR
CIHR
ZonMw
NHMRC
NIH
MRC
EU
Wellcome
Gates
ESRC
Spanish
Chinese

BMJ Open

How common is Patient and Public Involvement (PPI)? Cross-sectional analysis of frequency of PPI reporting in health research papers and associations with methods, funding sources, and other factors

Journal:	*BMJ Open*
Manuscript ID	bmjopen-2022-063356.R1
Article Type:	Original research
Date Submitted by the Author:	05-May-2022
Complete List of Authors:	Lang, Iain; University of Exeter<br>King, Angela; University of Exeter<br>Jenkins, Georgia; University of Exeter<br>Boddy, Kate; University of Exeter<br>Khan, Zohrah; University of Exeter<br>Liabo, Kristin ; University of Exeter Medical School, Patient and Public Involvement
**Primary Subject Heading**:	Research methods
Secondary Subject Heading:	Research methods
Keywords:	STATISTICS & RESEARCH METHODS, MEDICAL ETHICS, EPIDEMIOLOGY

SCHOLARONE™
Manuscripts

I, the Submitting Author has the right to grant and does grant on behalf of all authors of the Work (as defined in the below author licence), an exclusive licence and/or a non-exclusive licence for contributions from authors who are: i) UK Crown employees; ii) where BMJ has agreed a CC-BY licence shall apply, and/or iii) in accordance with the terms applicable for US Federal Government officers or employees acting as part of their official duties; on a worldwide, perpetual, irrevocable, royalty-free basis to BMJ Publishing Group Ltd ("BMJ") its licensees and where the relevant Journal is co-owned by BMJ to the co-owners of the Journal, to publish the Work in this journal and any other BMJ products and to exploit all rights, as set out in our licence.

The Submitting Author accepts and understands that any supply made under these terms is made by BMJ to the Submitting Author unless you are acting as an employee on behalf of your employer or a postgraduate student of an affiliated institution which is paying any applicable article publishing charge ("APC") for Open Access articles. Where the Submitting Author wishes to make the Work available on an Open Access basis (and intends to pay the relevant APC), the terms of reuse of such Open Access shall be governed by a Creative Commons licence – details of these licences and which Creative Commons licence will apply to this Work are set out in our licence referred to above.

Other than as permitted in any relevant BMJ Author's Self Archiving Policies, I confirm this Work has not been accepted for publication elsewhere, is not being considered for publication elsewhere and does not duplicate material already published. I confirm all authors consent to publication of this Work and authorise the granting of this licence.

For peer review only

How common is Patient and Public Involvement (PPI)? Cross-sectional analysis of frequency of PPI reporting in health research papers and associations with methods, funding sources, and other factors

Authors: Iain Lang[1], Angela King (A.King6@exeter.ac.uk)[1], Georgia Jenkins (G.Jenkins2@exeter.ac.uk)[1], Kate Boddy (K.Boddy@exeter.ac.uk)[1], Zohrah Khan (zohrah.khan@nhs.net)[1], Kristin Liabo (K.Liabo@exeter.ac.uk)[1]

Corresponding author:

a. Iain Lang
b. College of Medicine and Health, University of Exeter, UK
c. i.lang@exeter.ac.uk

Affiliations:

1 College of Medicine and Health, University of Exeter.

Abstract

Objectives. Patient and public involvement (PPI) in health research is required by some funders and publications but we know little about how common it is. In this study we estimated the frequency of PPI inclusion in health research papers and analysed how it varied in relation to research topics, methods, funding sources, and geographical regions.

Design. Cross-sectional

Methods. Our sample consisted of 3000 research papers published in 2020 in a general health-research journal (*BMJ Open*) that requires a statement on whether studies included PPI. We classified each paper as “included PPI” or “did not include PPI” and analysed the association of this classification with location (country or region of the world), methods used, research topic (journal section), and funding source. We used adjusted regression models to estimate incident rate ratios (IRRs) of PPI inclusion in relation to these differences.

Results. 618 (20.6%) of the papers in our sample included PPI. The proportion of papers including PPI varied in relation to location (from 44.5% (95% confidence interval (CI) 40.8 to 48.5) in papers from the UK to 3.4% (95% CI 1.5 to 5.3) in papers from China), method (from 38.6% (95% CI 27.1 to 50.1) of mixed-methods papers to 5.3% (95% CI -1.9 to 12.5) of simulation papers), topic (from 36.9% (95% CI 29.1 to 44.7) of papers on mental health to 3.4% (95% CI -1.3 to 8.2) of papers on medical education and training, and funding source (from 57.2% (95% CI 51.8% to 62.6%) in papers that received funding from the UK's National Institute for Health Research (NIHR) to 3.4% (95% CI 0.7% to 6.0%) in papers that received funding from a Chinese state funder).

Conclusions. Most research papers in our sample did not include PPI and PPI inclusion varied widely in relation to location, methods, topic, and funding source.

Word count (main text) = 3519

For peer review only

Keywords: patient and public involvement, PPI, patient engagement, stakeholder participation

For peer review only

Article Summary

Strengths and limitations of this study

- There has been little previous research on how often patient and public involvement (PPI) is included in health research papers and our sample (of 3000 papers) is much larger than that of previous studies
- As far as we are aware, this is the first study to analyse associations between PPI inclusion and other aspects of research papers such as topic and methods
- Our sample came from a single journal, but it is a general health- and medical-research journal that includes a broad range of topics and methods and requires authors to provide a statement on PPI inclusion
- We relied on published statements provided in research papers and cannot assess their accuracy

For peer review only

Background

The involvement and engagement of patients and members of the public in the conduct of health research has been endorsed and promoted by a range of government and other funders (1). The roots of this lie in the World Health Organization's Alma Alta Declaration (2), the product of a major international conference on public health that has had a lasting impact on health and healthcare, which stated that people have a "right and duty to participate individually and collectively in the planning and implementation of their healthcare." Those who promote patient and public involvement (PPI) in health research often argue for it based on one or both of two reasons, one normative and one practical(3). The normative reason is that PPI is seen as making research more democratic and egalitarian and to move activity towards research done *with* or *by* people rather than *on* or *about* them (4). The practical reason is that PPI is seen as benefiting not only the process of research, for example by improving recruitment and retention of participants in clinical trials (5), but also the quality of research and the impact of research findings (6–8). As a means of implementing PPI in health research, some research funders now recommend or stipulate patient and public involvement (PPI) in the work they fund. This is done by some state funders, like the UK's National Institute for Health Research (9) and the Australian National Health and Medical Research Council (10), as well as by charitable funders such as the UK's Alzheimer's Society (11) and McPin Foundation (12).

However, reviews of PPI have found low levels of involvement and poor-quality reporting (13–17). Even in areas where we might expect high levels of patient involvement, such as trials of patient-oriented research or the development of patient-reported outcome measures, PPI is often poorly reported or absent (18,19). Research into PPI has identified concerns amongst researchers grounded in perceptions of the costs involved (20), uncertainties about how to do it (21) or report it (22), and difficulties related to issues such as tokenism, box-ticking, and the role of PPI as a form of governance (23–27) .

To date, research on the inclusion of PPI has been almost entirely qualitative and we lack information on how widespread PPI is within health research and in what circumstances researchers are more or less likely to include it: information that is important if we are to understand and promote PPI. Our aim in this study was to provide some of this information. We used data on PPI in papers published in BMJ Open, an open-access journal in which PPI reporting is obligatory and authors are required to include a statement about PPI even if they did not include PPI in their study. We looked at all the PPI statements in all research papers published in BMJ Open in a twelve-month period. We analysed, first, how common PPI inclusion was and, second, whether and to what extent PPI varied in relation to location, method, topic, and funding source.

Methods

Data collection

We used ProQuest, an online bibliographic database (www.proquest.com), to identify all original research papers published in BMJ Open during the twelve months from 1st January 2020 to 31st December 2020 (n=3000), the most recent complete year for which data were available when we started data collection. The data for our study were the full texts of papers plus accompanying metadata (information on each paper detailing date of publication, document IDs, journal section, and so on). We used a custom-written Perl (28) script to read the data file, extract relevant information, and assemble our analysis dataset.

For peer review only

For each paper, we extracted:

- *First author name*, for ease of reference and reporting.
- the *URL* that linked directly to the online version of the paper using its digital object identifier (DOI) and which also constituted a unique identifier for each paper.
- *Patient and public involvement statement*: full text of the statement made in the paper (as part of the main text) or following the paper (alongside the acknowledgments).
- *Location* (country or region) from which the paper came, based on the country of the primary affiliation of the first author. Many countries were associated with only a small number of papers and for this reason, as well as for ease and clarity of reporting, we bundled all countries with fewer than 100 associated papers into regions. This left us with seven individual countries and six regions.
- *Method,* based on our categorization of words in the title, from which we identified 18 terms that allowed us to assign each paper to at least one category (systematic review, protocol, trial, etc.) in relation to methods. Some papers had more than one method associated with them because they contained multiple terms, e.g., both protocol and trial.
- *Research Topic,* based on the section of the journal to which the paper was assigned. Most of these related to substantive topics (Public Health, Cardiovascular Medicine, Nursing, etc.) and some relate to methods (Epidemiology, Qualitative research, Health Economics, etc.) but each paper is assigned to only one section. The papers in our sample fell into 61 sections but many of these had only small numbers of papers in them. For reporting purposes, below, we provide details of research topics from the 19 sections containing at least 50 papers.
- *Funding source*, extracted from the Funding section of each paper. We identified all funders associated with each paper but for reporting purposes we only provide details of those with at least 25 papers related to them: there were twelve of these. These more common funders were associated with approximately one-third of the papers in our sample.

We looked at four *exposure variables* based on this extracted information: location, method, research topic, and funding source. Our aim was to understand whether papers that differed in relation to these categories (differences in location, differences in methods, and so on) were also likely to differ in terms of including PPI.

We based our *outcome variable* on our interpretation of the PPI statement and categorized each paper as either "included PPI" or "did not include PPI". We used the Perl script to find phrases commonly used in PPI statements, such as "no patients involved", "patients and public were not involved", and "patients and/or the public were not invited". Based on this, the script assigned 1797 of the 3000 papers to the "did not include PPI" group. This assignation helped us reach our final decision on the group in which we should put each paper but did not constitute our decision: we read through each statement to check that we agreed with the categorization.

Deciding what does and does not constitute PPI is conceptually difficult, especially when only limited information – short statements provided by authors – is available. We classed papers as "included PPI" if any form of involvement by one or more non-professional

For peer review only

researchers, whether as individuals or in the form of a group, was claimed at any stage of the study. We classed as "did not include PPI" papers in which:

- PPI was explicitly omitted (e.g., "Patients and the public were not involved in this research study" (29)
- Patient input was taken from previous literature or previous studies (e.g., "Previous studies about patient experiences in OLP and placebo trials of irritable bowel syndrome were consulted for this study to include patient experiences" (30))
- PPI-type input was provided by experts whose primary role was not to provide knowledge from lived experience or public oversight of the study (e.g., "The indication, research questions and study endpoint outcome measures were selected based on the authors' expert understanding in the care of affected patients, their needs and therapy preferences, without direct communication of the study design to patients"(31).

One of the authors (IL) took responsibility for reading all the PPI statements, correcting the script-based assignments where they were wrong, and making a final decision on the categorization of each paper. IL and two other authors (KB and KL) did initial calibration for this decision-making in concert: all three of us discussed several borderline cases and arrived at a consensus on how to classify these. In 130 of the papers in our dataset we could not find a statement or any other information about PPI and we coded these as not including PPI.

Analysis

We analysed our data using Stata/SE 17.0 and Microsoft Excel. For each exposure category (location, method, research topic, funder) we calculated descriptive statistics relating to the number of paper assigned to each category and the mean level of PPI in each. For example, in relation to methods, did papers that reported trials, evaluations, or systematic reviews differ in terms of how likely they were to include PPI? What about papers that concerned surgery compared to those about anaesthetics or general practice? For the groupings within each category we estimated, with 95% confidence intervals, the proportion of papers in which the authors reported the inclusion of PPI.

In the regression models of which we report the results below, we used Stata to estimate the relative risk and confidence intervals of having PPI by means of generalized linear models with a modified Poisson approach and robust error variances (32). To account for potential confounding, we adjusted our models for location, method, and topic for each of the other exposure variables (location/ method/ topic/ funding source). When the outcome was funding source we adjusted for method and topic but not for location, because location and funding source were often overlapping. For example, of the 178 papers receiving funding from Chinese state funders, 174 (97.8%) came from China – see additional analyses below.

The highest and lowest levels of PPI inclusion were associated, respectively, with papers receiving funding from two national government funders, the UK's NIHR and Chinese state funders. To provide insight into the relationships with PPI inclusion of being based in a specific country and of being supported by the state funder in that country, we conducted additional analyses to look at the differences in PPI inclusion levels in UK papers that were and that were not funded by NIHR, and in Chinese papers that were and were not funded by Chinese state funders.

For peer review only

Ethics approval

Since this study used publicly available data and involved neither human nor animal subjects, we did not submit it for, and it has not received, formal ethics approval.

Patient and Public Involvement

One of our co-authors, AK, is not a professional researcher but has experience of being involved in research as a patient and carer. She is a member of the Peninsula Patient Engagement Group (PenPEG), has been a patient and carer for most of her life, and has remained active in both patient advocacy and PPI for over twenty-five years. She joined the study after inception and was involved in data analysis and interpretation and in writing the manuscript. From the perspective of the professional co-authors, AK's most important contribution has been to challenge us on how we have described PPI throughout the paper. She drew our attention to issues around language and power in an early draft and because of this we changed the way in which we described PPI throughout the paper.

Results

Of the 3000 papers in our sample, we identified 618 (20.6%, 95% CI 19.1% to 22.0%) as having included PPI. The tables and figures below show the distribution of these papers in relation to the four types of difference we examined: location (Table 1), method (Table 2), research topic (Table 3), and funder (Table 4). Each table shows the unadjusted estimates of mean PPI in each category, ordered from greatest to lowest:

- in relation to *location* (Table 1), the likelihood of PPI inclusion ranged from 44.5% (95% CI 40.5% to 48.5%) in the UK to 3.4% (95% CI 1.5% to 5.3%) in China.
- in relation to *method* (Table 2), the likelihood of PPI inclusion ranged from 38.6% (95% CI 27.1% to 50.1%) in mixed-methods papers to 5.3% (95% CI -1.9% to 12.5%) in simulation papers.
- in relation to *research topic* (Table 3), the likelihood of PPI inclusion ranged from 36.9% (95% CI 29.1% to 44.7%) in papers on mental health to 3.4% (95% CI -1.3% to 8.2%) of papers on medical education and training.
- in relation to *funding source* (Table 4), the likelihood of PPI inclusion ranged from 57.2% (95% CI 51.8% to 62.6%) in papers that received funding from NIHR to 3.4% (95% CI 0.7% to 6.0%) in papers that received funding from a Chinese state funder.

Papers that did not receive funding from any of the major funders (that is, those included in Table 4) (n=2028) had a mean level of PPI inclusion of 14.9% (95% confidence interval 13.4% to 16.5%), which is statistically significantly lower than the mean level of PPI inclusion of the whole dataset.

Figures 1, 2, 3, and 4 show the relative risks and confidence intervals of each category estimated using an adjusted generalized linear model as described above for location, method, research topic, and funding source, respectively.

For peer review only

Table 1. Number of papers published in BMJ Open in 2020 by location, and proportion of these with PPI

Location	n	% of total	Mean %PPI (95% CI)
UK	591	19.7	44.5 (40.5 to 48.5)
Canada	195	6.5	30.8 (24.3 to 37.3)
Australia/NZ	269	9.0	21.9 (17.0 to 26.9)
France	118	3.9	21.2 (13.8 to 28.6)
Netherlands	120	4.0	20.8 (13.5 to 28.1)
Germany	101	3.4	16.8 (9.5 to 24.2)
USA	227	7.6	16.3 (11.5 to 21.1)
Europe (other)	501	16.7	15.5 (12.4 to 18.7)
Central and South America	59	2.0	11.3 (3.9 to 18.7)
Central and South Asia	72	2.4	10.2 (2.4 to 18.0)
Africa and West Asia	163	5.4	8.0 (3.8 to 12.1)
East and South-east Asia	232	7.7	6.4 (3.3 to 9.6)
China	352	11.7	3.4 (1.5 to 5.3)
	3000	100.0	20.6 (19.1 to 22.0)

Note that location here means each country with ≥100 papers and otherwise means region

Table 2. Number of papers published in BMJ Open in 2020 by method used, and proportion of these with PPI

Method	n	% of total	Mean %PPI (95% CI)
Mixed methods	70	2.3	38.6 (27.1 to 50.1)
Implementation	65	2.2	35.4 (23.7 to 47.1)
Trial	551	18.4	34.8 (30.9 to 38.8)
Qualitative	260	8.7	34.2 (28.4 to 40.0)
Evaluation	115	3.8	33.0 (24.4 to 41.7)
Pilot study	42	1.4	31.0 (16.8 to 45.1)
Development	118	3.9	29.7 (21.3 to 37.9)
Protocol	1181	39.3	28.9 (26.3 to 31.5)
Health economics	91	3.0	25.3 (16.3 to 34.3)
Survey	158	5.3	17.7 (11.7 to 23.7)
Observational	220	7.3	15.9 (11.1 to 20.8)
Cohort	637	21.2	14.6 (11.9 to 17.3)
Scoping	125	4.2	13.6 (7.6 to 19.6)
Time series	25	0.8	12.0 (-1.0 to 25.0)
Systematic review	376	12.5	9.6 (6.6 to 12.6)
Cross-sectional	464	15.5	9.3 (6.6 to 11.9)
Meta-analysis	265	8.9	8.3 (5.0 to 11.6)
Simulation	38	1.3	5.3 (-1.9 to 12.5)

Note that total n and % of papers here each sum to more than the total number of papers or to >100% because some have more than one designation – e.g., both protocol and trial

For peer review only

Table 3. Number of papers published in BMJ Open in 2020 in each research topic with ≥50 papers, and proportion of these with PPI

Research topic	n	% of total	Mean %PPI (95% CI)
Mental health	149	5.0	36.9 (29.1 to 44.7)
Qualitative research	51	1.7	35.3 (22.0 to 48.5)
Health services research	168	5.6	31.0 (23.9 to 38.0)
Neurology	82	2.7	29.3 (19.4 to 39.2)
Surgery	102	3.4	28.4 (19.6 to 37.2)
Rehabilitation medicine	65	2.2	26.2 (15.4 to 36.9)
Oncology	88	2.9	25.0 (15.9 to 34.1)
Cardiovascular medicine	120	4.0	24.2 (16.5 to 31.9)
Paediatrics	91	3.0	23.1 (14.4 to 31.8)
Diabetes and endocrinology	85	2.8	22.4 (13.4 to 31.3)
Obstetrics and gynaecology	98	3.3	21.4 (13.3 to 29.6)
Emergency medicine	66	2.2	21.2 (11.3 to 31.2)
General practice / Family practice	94	3.1	20.2 (12.0 to 28.4)
Public health	366	12.2	16.1 (12.3 to 19.9)
Infectious diseases	79	2.6	15.2 (7.2 to 23.2)
Global health	97	3.2	13.4 (6.6 to 20.2)
Health economics	61	2.0	6.6 (0.3 to 12.8)
Epidemiology	241	8.0	6.2 (3.2 to 9.3)
Medical education and training	58	1.9	3.4 (-1.3 to 8.2)
	1261	71.8	

Table 4. Number of papers published in BMJ Open in 2020 by funding source for funders named in ≥25 papers, and proportion of these with PPI

Funder	n	% of total	Mean %PPI (95% CI)
NIHR	320	10.7	57.2 (51.8 to 62.6)
CIHR	90	3.0	41.1 (30.9 to 51.3)
ZonMw	25	0.8	40.0 (20.4 to 59.6)
MRC	58	1.9	39.7 (27.0 to 52.4)
ESRC	41	1.4	29.3 (15.2 to 43.4)
Wellcome Trust	103	3.4	28.2 (19.4 to 36.9)
NHMRC	111	3.7	27.9 (19.5 to 36.3)
NIH	81	2.7	27.2 (17.4 to 36.9)
EU	69	2.3	23.2 (13.2 to 33.2)
Carlos III	30	1.0	13.3 (1.0 to 25.7)
Gates Foundation	33	1.1	12.1 (0.8 to 23.4)
Chinese	178	5.9	3.4 (0.7 to 6.0)

Note: NIHR = National Institute of Health Research (UK); ZonMw = The Netherlands Organisation for Health Research and Development; CIHR = Canadian Institutes of Health Research; MRC = Medical Research Council (UK); ESRC = Economic and Social Research Council (UK); NHMRC = National Health and Medical Research Council (Australia); Wellcome = The Wellcome Trust (UK); EU = European Union funding; NIH = US National Institutes of Health; Carlos III = Instituto de Salud Carlos III (Spain); Gates = The Bill and Melinda Gates Foundation (USA); Chinese = Key Research or other Chinese state funder, e.g. National Science Foundation of China)

For peer review only

Additional analyses

Of the 320 papers whose authors reported receiving funding from the UK's NIHR, 272 (85.0%) came from the UK. Those 272 papers represent 46.0% of the 591 UK papers in our sample, and 59.9% (95% CI 54.1% to 65.7%) of them included PPI. Of the 319 (54.0%) of UK papers whose authors did not report receiving NIHR funding, 31.3% (95% CI 26.2% to 36.5%) included PPI. For UK papers, receipt of NIHR funding was associated with increased PPI inclusion.

Of the 178 papers whose authors reported receiving funding from Chinese state funders, 174 (97.8%) came from China. Those 174 papers represent 49.3% of the 352 Chinese papers in our sample, and 3.4% (95% CI 0.7% to 6.2%) of them included PPI. Of the 178 (50.6%) of Chinese papers whose authors did not report receiving Chinese state funding, 3.4% (95% CI 0.7% to 6.0%) included PPI. For Chinese papers, receipt of Chinese state funding was not associated with any difference in PPU inclusion.

Discussion

We had two aims in this study: to assess how common PPI is in a sample of published health research papers, and to measure how much variation there is in PPI inclusion according to known differences between the papers. We addressed these aims by looking the PPI statements included in 3000 papers published in general health and medical research journal where authors are required to include a statement about PPI, BMJ Open. In relation to our first aim, we found that approximately one in five (20.6%) of the papers in our sample reported including PPI. In relation to the second, we found marked variations in PPI inclusion across the categories we looked at. For example, papers from the UK were more than ten times likelier to include PPI than those from China, and there were also marked differences in relation to each of method, topic, and funding source.

These large differences may indicate where there is potential to increase PPI inclusion in research. For example, funders wishing to increase PPI inclusion might target support and promotion to researchers working in fields with low levels of inclusion. The funding source we found to be associated with the highest level of PPI inclusion was the UK's National Institute for Health Research (NIHR), which has for several years included in its application process a section in which applicants may either apply for funding to include PPI or justify why they are not including it. We note, at the same time, that NIHR lacks a single, unequivocal policy statement about PPI (33).

Previous work has reviewed PPI reporting in specific topics such as dementia (14,34), patient-oriented-research trials (18), and surgical trials (15). Although the authors of these studies did not attempt to measure the proportion of published papers involving PPI, as we have done here, they uniformly identify few studies reporting PPI within the large literatures in which they were interested; a study of clinical trials published in nursing journals failed to find any evidence of PPI (35). We know of only one recent study on the prevalence of PPI in general medical research, which, in a smaller sample, found that 11% of papers reported PPI inclusion (36). We are unaware of any other studies on the prevalence of PPI inclusion or of factors associated with it.

Our study has some noteworthy limitations. The first of these relates to PPI statements. We took authors' PPI statements at face value: if the statement said that PPI took place then we took this to be the case, even though there is evidence that what researchers say they will do and what they actually do, in relation to PPI, varies (37). Authors may have exaggerated, underplayed, or otherwise misreported the extent of PPI in their studies and the findings we

For peer review only

have reported do not allow us to say anything about the quality of the PPI in the "included PPI" studies. We had no way of telling whether PPI statements reflect the experience of involvement of the individuals or groups providing the PPI and note that statements about participation and involvement are often performative (38) and always political (39). However, we note the conclusion of a previous study: that apparently low levels of PPI inclusion are not simply due to under-reporting (36).

A second, related, limitation is that PPI may be understood differently in different places and, as a result, reported differently. We found PPI is more common in health research from the UK than elsewhere. The development and institutionalization of PPI in the UK can be seen as the outcome of specific national socio-political events and trends. As a result, the term "patient and public involvement" has resonances in UK health research that may be different or absent elsewhere. As a group of authors, we are most familiar with policies and expectations around PPI in the country in which we are based, the UK, so we cannot comment in detail on the ways PPI may be viewed or implemented elsewhere. In other settings there may be other ways of addressing the concerns that PPI is seen as addressing (for example, through working with patient-interest organizations rather than with individual patient representatives) and a focus on PPI may overlook these. A recent systematic review of attitudes and approaches to PPI in Europe (40) found a convergence of understandings in some European nations but lack of institutional support in many places. Although not focusing on research, Zhu (41) describes how patient involvement and participation in Chinese hospitals is influenced by factors including level of civil society engagement, moves towards marketisation, and individuals' cultural resources. Possibilities for and meanings of PPI may vary widely in relation to both national and sub-national differences and the differences may be subtle: Fang (42) has written about the challenges he, a Chinese immigrant to the US, faces when preparing scholarly works in English, which has an entirely different set of norms and expectations related to how scholars should write and argue.

A third limitation relates to our sample. We used a purposeful sample of papers from a single journal, BMJ Open – if this were a clinical study we might call it a case series. We used BMJ Open because, as far as we know, it is the only journal that (a) has a written policy requiring papers to include a PPI statement *and* (b) is open access *and* (c) is not discipline- or method-specific and publishes papers across a range of health-related topics and range of methods. Using only a single journal also simplified our analysis, since we did not have to consider or attempt to account for variations across journals in terms of editorial policy or other factors. However, peer-reviewed journals are, by definition, selective in the papers in they publish. BMJ Open publishes many study protocols and many papers by authors based in the UK. The journal's policy regarding PPI may encourage authors to submit papers that include PPI and discourage them from submitting those that lack it. These characteristics may influence the topics and methods submitted to the journal as well as actions concerning PPI.

We believe there is scope for further research on the issues we have begun to explore here. This might include work on the quality of PPI reporting, perhaps based on established criteria; replication of our analyses using a sample of papers taken from elsewhere; and qualitative analysis of the statements made both by authors who report PPI and those who report none. Our dichotomisation of statements into "included PPI" or "did not include PPI" ignores any issues of quality or extent of involvement and this topic merits investigation. Our analysis has looked only at associations between PPI involvement and broad categories such as country of origin or funding body and not micro-level differences such as attitudes, policies, or beliefs at the level of research organisations, research groups, or individual researchers. We will need different methods to study such influences on how and why PPI in health research occurs – and why it does not.

For peer review only - http://bmjopen.bmj.com/site/about/guidelines.xhtml

Contributorship Statement: Concept and design, statistical analysis, and drafting of the manuscript: IL. Acquisition, analysis, or interpretation of the data, critical revision of the manuscript for important intellectual content, approval of final version, and agreement to be accountable for all aspects of the work: IL, AK, GJ, KB, ZK, KL.

Competing interests: None declared

Funding: This report is independent research supported by the UK National Institute for Health Research Applied Research Collaboration South West Peninsula (Grant Reference Number: NIHR200167). The views expressed in this publication are those of the author(s) and not necessarily those of the National Institute for Health Research or the UK Department of Health and Social Care.

Data sharing statement: Data for this study came from an open-source journal and are freely available online

For peer review only

References

1. Hoddinott P, Pollock A, O'Cathain A, Boyer I, Taylor J, MacDonald C, et al. How to incorporate patient and public perspectives into the design and conduct of research. F1000Research. 2018;7:752.

2. World Health Organisation. Declaration of Alma Ata: report of the International Conference on Primary Health Care. In Geneva; 1978.

3. Ives J, Damery S, Redwod S. PPI, paradoxes and Plato: who's sailing the ship? J Med Ethics. 2013 Mar;39(3):181–5.

4. Heron J, Reason P. Extending Epistemology within a Co-operative Inquiry. In: The SAGE Handbook of Action Research [Internet]. 1 Oliver's Yard, 55 City Road, London England EC1Y 1SP United Kingdom: SAGE Publications Ltd; 2008 [cited 2022 Mar 11]. p. 366–80. Available from: http://methods.sagepub.com/book/the-sage-handbook-of-action-research/d32.xml

5. Crocker JC, Ricci-Cabello I, Parker A, Hirst JA, Chant A, Petit-Zeman S, et al. Impact of patient and public involvement on enrolment and retention in clinical trials: systematic review and meta-analysis. BMJ. 2018 Nov 28;363:k4738.

6. Staley K. Exploring impact: public involvement in NHS, public health and social care research. Eastleigh, England: INVOLVE; 2009.

7. Evans D, Coad J, Cottrell K, Dalrymple J, Davies R, Donald C, et al. Public involvement in research: assessing impact through a realist evaluation [Internet]. Southampton (UK): NIHR Journals Library; 2014 [cited 2022 Mar 11]. (Health Services and Delivery Research). Available from: http://www.ncbi.nlm.nih.gov/books/NBK260168/

8. Crocker JC, Boylan A, Bostock J, Locock L. Is it worth it? Patient and public views on the impact of their involvement in health research and its assessment: a UK-based qualitative interview study. Health Expect Int J Public Particip Health Care Health Policy. 2017 Jun;20(3):519–28.

9. UK Public Involvement Standards Development Partnership. UK Standards for Public Involvement: Better public involvement for better health and social care research [Internet]. [cited 2022 Mar 18]. Available from: https://drive.google.com/file/d/1U-IJNJCfFepaAOruEhzz1TdLvAcHTt2Q/view

10. Consumers Health Forum of Australia. Statement on consumer and community involvement in health and medical research. National Health and Medical Research Council; 2016.

11. Alzheimer's Society. Alzheimer's Society Research Delivery Plan 2018/2022 [Internet]. 2017 [cited 2022 Mar 14]. Available from: https://www.alzheimers.org.uk/sites/default/files/2019-05/AS_ResearchDeliveryPlan_2019_v6final.pdf

12. McPin Foundation. Patient & Public Involvement at the McPin Foundation: An overview. Version 1.1, July 2017 [Internet]. 2017 [cited 2022 Mar 14]. Available from: https://mcpin.org/wp-content/uploads/McPin-PPI-slides-v1.1-july-2017.pdf

13. Cook N, Siddiqi N, Twiddy M, Kenyon R. Patient and public involvement in health research in low and middle-income countries: a systematic review. BMJ Open. 2019 May 1;9(5):e026514.

14. Miah J, Dawes P, Edwards S, Leroi I, Starling B, Parsons S. Patient and public involvement in dementia research in the European Union: a scoping review. BMC Geriatr. 2019 Aug 14;19(1):220.

15. Jones EL, Williams-Yesson BA, Hackett RC, Staniszewska SH, Evans D, Francis NK. Quality of Reporting on Patient and Public Involvement Within Surgical Research: A Systematic Review. Ann Surg. 2015 Feb;261(2):243–50.

16. Muir R, Carlini JJ, Harbeck EL, Gillespie BM, Tuffaha HW, Walker RM, et al. Patient involvement in surgical wound care research: A scoping review. Int Wound J. 2020;17(5):1462–82.

17. Patel VA, Shelswell J, Hillyard N, Pavitt S, Barber SK. A study of the reporting of patient and public involvement and engagement (PPIE) in orthodontic research. J Orthod. 2021 Mar 1;48(1):42–51.

18. Fergusson D, Monfaredi Z, Pussegoda K, Garritty C, Lyddiatt A, Shea B, et al. The prevalence of patient engagement in published trials: a systematic review. Res Involv Engagem. 2018 May 22;4(1):17.

19. Wiering B, de Boer D, Delnoij D. Patient involvement in the development of patient-reported outcome measures: a scoping review. Health Expect. 2017;20(1):11–23.

20. Baldwin JN, Napier S, Neville S, Wright-St Clair VA. Impacts of older people's patient and public involvement in health and social care research: a systematic review. Age Ageing. 2018 Nov 1;47(6):801–9.

21. Domecq JP, Prutsky G, Elraiyah T, Wang Z, Nabhan M, Shippee N, et al. Patient engagement in research: a systematic review. BMC Health Serv Res. 2014 Feb 26;14(1):89.

22. Price A, Albarqouni L, Kirkpatrick J, Clarke M, Liew SM, Roberts N, et al. Patient and public involvement in the design of clinical trials: An overview of systematic reviews. J Eval Clin Pract. 2018;24(1):240–53.

23. Komporozos-Athanasiou A, Renedo A, McKevitt C. Citizen Participation in Neoliberal Times. Sociol Res Online. 2019 Sep 1;24(3):370–5.

24. Komporozos-Athanasiou A, Paylor J, Mckevitt C. Governing Researchers through Public Involvement. J Soc Policy. 2021 Apr 16;1–16.

25. Sangill C, Buus N, Hybholt L, Berring LL. Service user's actual involvement in mental health research practices: A scoping review. Int J Ment Health Nurs. 2019;28(4):798–815.

26. Boaz A, Biri D, McKevitt C. Rethinking the relationship between science and society: Has there been a shift in attitudes to Patient and Public Involvement and Public Engagement in Science in the United Kingdom? Health Expect. 2016;19(3):592–601.

27. Lemonsky F. Service user involvement in research. Lancet Psychiatry. 2015 Sep 1;2(9):780.

28. Wall L, Christiansen T, Schwartz R. Programming Perl. 3rd edition. USA: O'Reilly Media, Inc; 2000. 670 p.

29. Alahdab F, Halvorsen AJ, Mandrekar JN, Vaa BE, Montori VM, West CP, et al. How do we assess resilience and grit among internal medicine residents at the Mayo Clinic? A longitudinal validity study including

For peer review only

correlations with medical knowledge, professionalism and clinical performance. BMJ Open. 2020 Dec 1;10(12):e040699.

30. Nascimento AF, Gaab J, Kirsch I, Kossowsky J, Meyer A, Locher C. Open-label placebo treatment of women with premenstrual syndrome: study protocol of a randomised controlled trial. BMJ Open. 2020 Feb 1;10(2):e032868.

31. Vincenzi B, Trower M, Duggal A, Guglielmini P, Harris P, Jackson D, et al. Neurokinin-1 antagonist orvepitant for EGFRI-induced pruritus in patients with cancer: a randomised, placebo-controlled phase II trial. BMJ Open. 2020 Feb 1;10(2):e030114.

32. Zou G. A Modified Poisson Regression Approach to Prospective Studies with Binary Data. Am J Epidemiol. 2004 Apr 1;159(7):702–6.

33. Russell J, Greenhalgh T, Taylor M. Patient and public involvement in NIHR research 2006-2019: policy intentions, progress and themes. :37.

34. Bethell J, Commisso E, Rostad HM, Puts M, Babineau J, Grinbergs-Saull A, et al. Patient engagement in research related to dementia: A scoping review. Dementia. 2018 Nov 1;17(8):944–75.

35. Gray R, Brasier C, Zirnsak TM, Ng AH. Reporting of patient and public involvement and engagement (PPIE) in clinical trials published in nursing science journals: a descriptive study. Res Involv Engagem. 2021 Dec 14;7(1):88.

36. Price A, Schroter S, Snow R, Hicks M, Harmston R, Staniszewska S, et al. Frequency of reporting on patient and public involvement (PPI) in research studies published in a general medical journal: a descriptive study. BMJ Open. 2018 Mar 1;8(3):e020452.

37. Blackburn S, McLachlan S, Jowett S, Kinghorn P, Gill P, Higginbottom A, et al. The extent, quality and impact of patient and public involvement in primary care research: a mixed methods study. Res Involv Engagem. 2018 May 24;4(1):16.

38. Rosamund Snow: What makes a real patient? [Internet]. The BMJ. 2016 [cited 2022 Apr 29]. Available from: https://blogs.bmj.com/bmj/2016/07/19/rosamund-snow-what-makes-a-real-patient/

39. White SC. Depoliticising development: The uses and abuses of participation. Dev Pract. 1996 Feb;6(1):6–15.

40. Biddle MSY, Gibson A, Evans D. Attitudes and approaches to patient and public involvement across Europe: A systematic review. Health Soc Care Community. 2021;29(1):18–27.

41. Zhu J. The Meaning of Patient Involvement and Participation in Chinese Hospitals [Internet]. University of Edinburgh; 2021. Available from: https://era.ed.ac.uk/bitstream/handle/1842/37848/Zhu2021.pdf

42. Fang Z. Demystifying Academic Writing: Genres, Moves, Skills, and Strategies. New York: Routledge; 2021. 286 p.

For peer review only

Figure 1. Relative risk of PPI (incidence-rate-ratio) in studies by location compared to most common to UK, with 95% confidence intervals, adjusted by method, topic, and funder

Note: Incidence-rate ratios (y-axis) are on a logarithmic scale (base 10). Location here means each country with ≥100 papers and otherwise means region

Figure 2. Relative risk (incidence-rate ratio) of PPI in studies by method, with 95% confidence intervals, adjusted by location, topic, and funder

Note: Incidence-rate ratios (y-axis) are on a logarithmic scale (base 10)

Figure 3. Relative risk (incidence-rate ratio) of PPI in studies by topic compared to Health Service Research, with 95% confidence intervals, adjusted by location, method, and funder

Note: Incidence-rate ratios (y-axis) are on a logarithmic scale (base 10)

Figure 4. Relative risk (incidence-rate ratio) of PPI in studies by funding source compared to mean level, with 95% confidence intervals, adjusted by method and topic

Note: NIHR = National Institute of Health Research (UK); ZonMw = The Netherlands Organisation for Health Research and Development; CIHR = Canadian Institutes of Health Research; MRC = Medical Research Council (UK); ESRC = Economic and Social Research Council (UK); NHMRC = National Health and Medical Research Council (Australia); Wellcome = The Wellcome Trust (UK); EU = European Union funding; NIH = US National Institutes of Health; Carlos III = Instituto de Salud Carlos III (Spain); Gates = The Bill and Melinda Gates Foundation (USA); Chinese = Key Research or other Chinese state funder, e.g. National Science Foundation of China). Incidence-rate ratios (y-axis) are on a logarithmic scale (base 10)

For peer review only

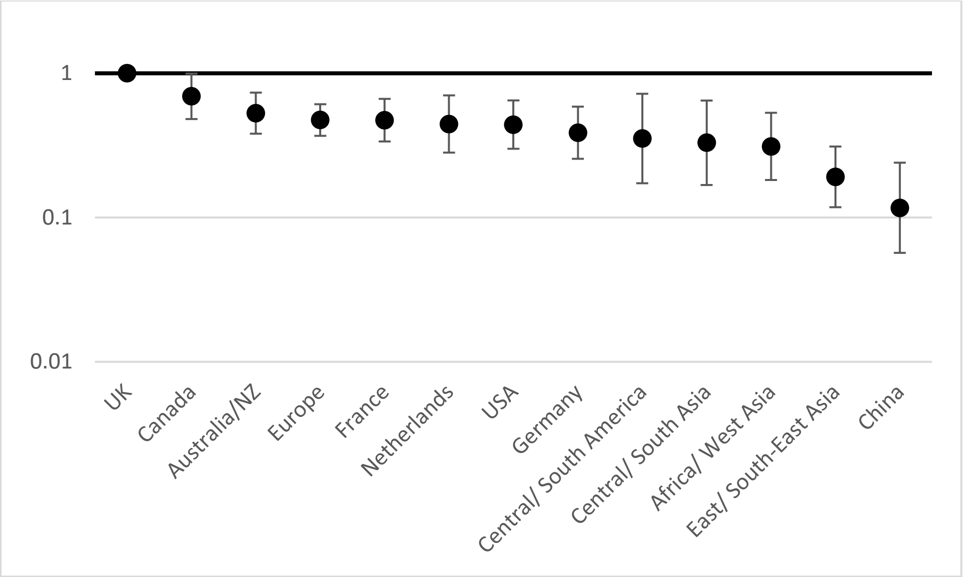
1
0.1
0.01
UK
Canada
Australia/NZ
Europe
France
Netherlands
USA
Germany
Central/ South America
Central/ South Asia
Africa/ West Asia
East/ South-East Asia
China

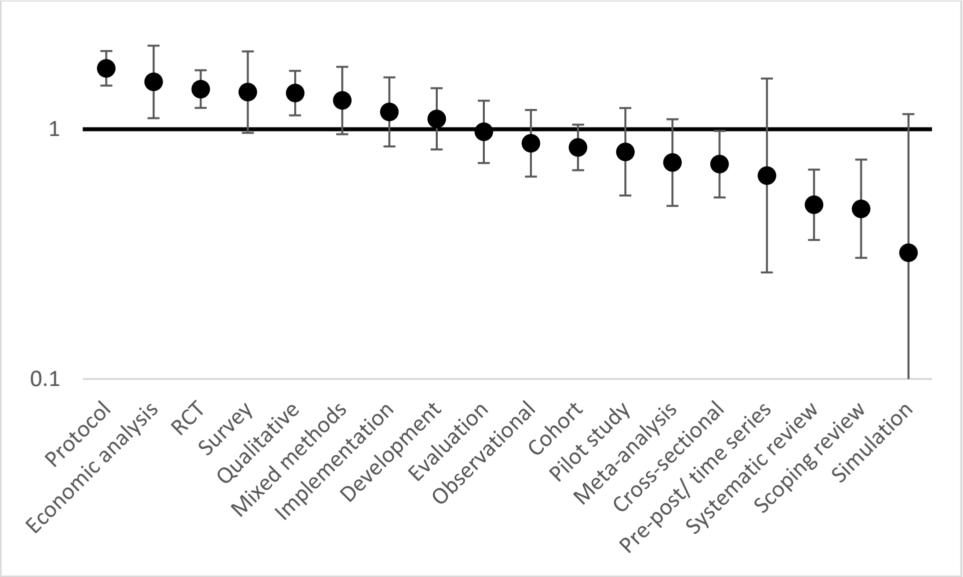

For peer review only

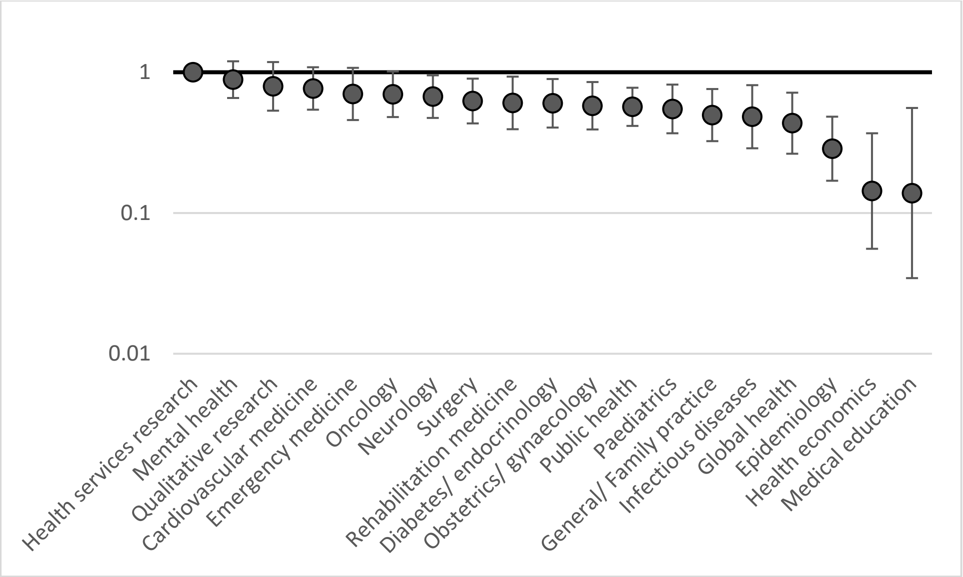

For peer review only

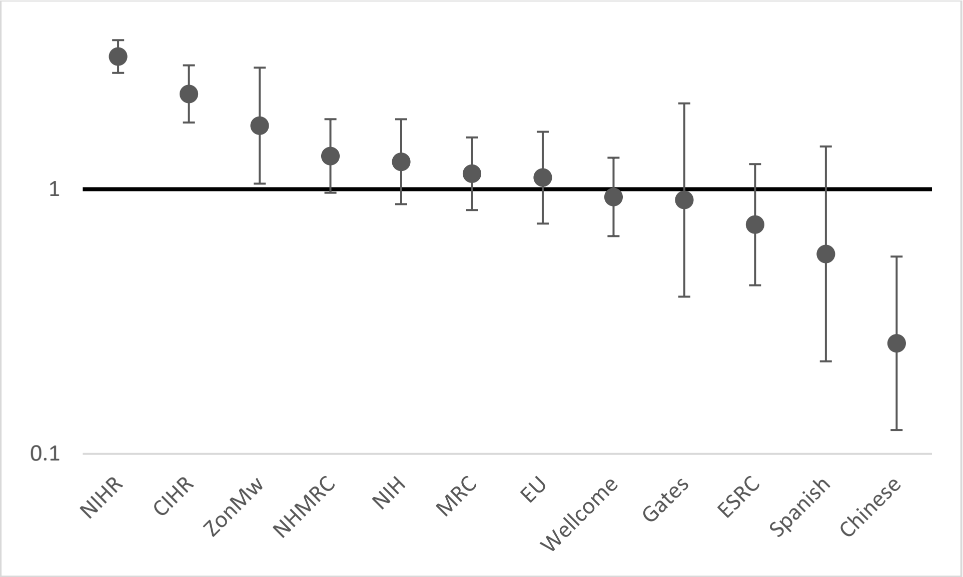

For peer review only
